# Chronic Treatment with the GLP1 Analogue Liraglutide Increases Cell Proliferation and Differentiation into Neurons in an AD Mouse Model

**DOI:** 10.1371/journal.pone.0058784

**Published:** 2013-03-11

**Authors:** Vadivel Parthsarathy, Christian Hölscher

**Affiliations:** School of Biomedical Sciences, Ulster University, Coleraine, United Kingdom; Universidad de Sevilla, Spain

## Abstract

Neurogenesis is a life long process, but the rate of cell proliferation and differentiation decreases with age. In Alzheimer's patients, along with age, the presence of Aβ in the brain inhibits this process by reducing stem cell proliferation and cell differentiation. GLP-1 is a growth factor that has neuroprotective properties. GLP1 receptors are present on neuronal progenitor cells, and the GLP-1 analogue liraglutide has been shown to increase cell proliferation in an Alzheimer's disease (AD) mouse model. Here we investigated acute and chronic effects of liraglutide on progenitor cell proliferation, neuroblast differentiation and their subsequent differentiation into neurons in wild type and APP/PS-1 mice at different ages. APP/PS1 and their littermate controls, aged 3, 6, 12, 15 months were injected acutely or chronically with 25 nmol/kg liraglutide. Acute treatment with liraglutide showed an increase in cell proliferation in APP/PS1 mice, but not in controls whereas chronic treatment increased cell proliferation at all ages (BrdU and Ki67 markers). Moreover, numbers of immature neurons (DCX) were increased in both acute and chronic treated animals at all ages. Most newly generated cells differentiated into mature neurons (NeuN marker). A significant increase was observed with chronically treated 6, 12, 15 month APP/PS1 and WT groups. These results demonstrate that liraglutide, which is currently on the market as a treatment for type 2 diabetes (Victoza^TM^), increases neurogenesis, which may have beneficial effects in neurodegenerative disorders like AD.

## Introduction

Adult neurogenesis is a process of continuous generation of new neurons and glia cells from neuronal progenitor/stem cell (NSC), which incorporates in existing circuitry [1]. Neurogenesis in the mammalian brain arises from the subventricular zone (SVZ) in the lateral ventricles, and the subgranular zone (SGZ) in the hippocampal dentate gyrus [2]–[4]. Adult neurogenesis is believed to be associated with memory, learning and the facilitation of long term potentiation of synaptic transmission [5]. Factors regulating adult neurogenesis include stress, age, inflammation processes and drugs [6]–[9]. A decrease in hippocampal neurogenesis is a natural ageing process [10], [11], which is exacerbated in pathological conditions like Parkinson's and Alzheimer's disease AD [12], [13]. There is the potential that the normalisation of stem cell proliferation in the brain may be of benefit in treating neurodegenerative diseases, and therefore, this area of research has been a focus point [14]–[17].

In AD, proliferation of NSCs, differentiation and neuronal survival are adversely affected [18], and this is linked to the accumulation of amyloid plaques [19] and the induction of chronic inflammation [20], [21]. Growth factors such as insulin activate stem cell proliferation [22], and recently, it has been found that insulin signaling in the brains of AD patients is desensitised [23]–[25]. Impaired insulin signalling furthermore has clear detrimental effects on cognition and on amyloid production [26], [27]. Pharmacological stimulation to increase proliferation of endogenous NSC and their subsequent differentiation to new neurons might serve as therapeutics for central nervous system disorders with neurodegeneration as common characteristic feature.

Glucagon like peptide -1 (GLP-1) is an endogenous incretin hormone of 30-amino acid produced by the intestinal L cells [28]. Liraglutide (Victoza®), a GLP-1 analogue with an extended half-life is used in type 2 diabetes treatment [29]. Subcutaneous injections of liraglutide in humans are well tolerated without affecting blood glucose levels in normoglycemic individuals [30].

Liraglutide displays glucoregulatory affects, facilitates insulin secretion during periods of hyper-glycaemia and increases beta cell mass [29], [31]. GLP-1R is expressed in hypothalamus, hippocampus and neurons [32], [33]. GLP-1, liraglutide and other GLP-1 analogues cross the blood brain barrier [34]–[36] where they specifically bind to GLP-1R to improve learning, memory and exert neuroprotective effects [35], [37]. Furthermore, enhanced learning and memory was observed in wild-type mice with increased expression of hippocampal GLP-1R [38] whereas GLP-1R knockout animals showed learning deficiencies [32], [38]. Previously, liraglutide has been shown to increase LTP [35], [39]–[41], reduce beta amyloid oligomers, plaque load, chronic inflammation and increase synaptic numbers in APP/PS1 mice [35].

In this study, effects of acute and long-term systemic administration of liraglutide on cell differentiation in wild type and APP/PS1 mice at different ages were examined. We have previously shown that liraglutide increases neural progenitor/stem cell proliferation [42] and now investigate if cells survive and differentiate into neuronal phenotype in the dentate gyrus of the mouse brain.

## Materials and Methods

### 2.1. Animals

Forty-eight APP*_SWE_*/PS1_ΔE9_ and Forty-eight wild type littermate female mice aged 3, 6, 12, 15 were used in the experiment. They were housed in single cages in a temperature controlled holding room (21.5°C±1) with 12∶12 hour light and dark cycle. Food and water were provided *ad libitum*. All experiments were carried out in accordance with the UK animals (Scientific Procedures) Act 1986.

### 2.2. Drug Treatment

Control group (n = 6) and liraglutide treated group (n = 6) were injected intraperitonially once daily with 0.9% saline or 25 nmol/kg body weight liraglutide (GL Biochem Ltd Shanghai) respectively for 7 days (acute treatment) and 37 days (chronic treatment). 5-bromo-2′-deoxyuridine (BrdU, 50 mg/kg i.p. Sigma, UK) is a thymidine analogue, which binds to DNA during S-phase and is used as cell proliferation marker was injected once daily for 7 days with the drug or saline. Animals were culled after 30 days after last BrdU injection.

### 2.3. Perfusion

All animals were transcardially perfused with ice-cold phosphate buffer. Brains were then retrieved and was placed in ice cold 4% paraformaldehyde. After 24 hours brains were transferred to 30% sucrose overnight, snap frozen with Envirofreez^TM^ (Sigma, UK) and 40 microns thick coronal sections were cut at anatomical regions of −2 to −3 bregma [43] using Leica cryostat. The first section was taken at random, then every 5^th^ section afterwards [44] was preserved in cryoprotect.

### 2.4. Immunohistochemistry

For BrdU and Doublecortin staining, endogenous peroxidase activity was quenched by treating sections with 3% H2O2. Sections were incubated in 0.3% Triton -100X and after washing with PBS, DNA denaturation for BrdU staining was carried out by incubating sections in 2N HCl at 37°C and then all sections were blocked in 5% normal serum. Sections were then incubated in monoclonal mouse anti BrdU (1∶200, B8434, Sigma Aldrich, USA) or goat polyclonal anti doublecortin (1∶200, sc-8066, SantaCruz, Germany) and incubated overnight at 4°C. Sections were then washed with phosphate buffer saline and biotinylated secondary antibody (1∶200 anti mouse for BrdU or 1∶500 anti goat for Doublecortin) was added. This was followed by amplification with Avidin-Biotin complex (Vectastain elite ABC kit PK-6102 and PK-6105, Vector laboratories Ltd, Peterborough, UK) and visualized using a detection kit (SK-4700, SG Substrate, Vector laboratories Ltd, Peterborough, UK).

For Ki67 staining, sections were treated with 10 mM sodium citrate buffer at 90°C for 30 minutes for antigen retrieval and after incubating in 0.3% triton-100X, sections were blocked in 5% serum and 5% BSA mixture and then incubated in rabbit polyclonal anti Ki67 antibody (1∶200, ab15580, Abcam, UK) overnight at 4°C. Sections were then washed and incubated in fluorescent secondary Alexa Fluor® goat anti rabbit 488 antibody (1∶200, A11008, Invitrogen, UK) and mounted on slide.

### 2.5. Double Immunoflourescence

To determine the phenotype of newly generated cells double Immunoflourescence was used. Tissue sections were permeabilized in 0.3% Triton -100X, denatured in 2N HCl and after neutralisation with 0.1M Borate buffer were blocked 5% goat serum+ 5% BSA. Sections were then incubated overnight at 4°C in mixture of primary antibodies of either monoclonal rat anti BrdU (1∶200, AB6326, Abcam, Cambridge, UK) and mouse monoclonal anti NeuN (mature neuron marker, 1∶200, MAB377, Millipore, USA) or monoclonal mouse anti GFAP (astrocyte marker, 1∶500, MAB3402, Millipore) or polyclonal rabbit anti Iba1 (microglia marker, 1∶1000, 001-20001, Wako, Germany). Sections were washed in PBS and incubated for 60 min at room temperature in mixture of fluorescent secondary antibodies of goat anti rat Alexa Fluor® 555 (1∶150, A21434, Invitrogen, UK) with goat anti mouse Alexa Fluor® 488 (1∶150, A11001) goat anti rabbit Alexa Fluor® 488 (1∶150 A11008, Invitrogen, UK). Sections were mounted on silanized slides and cover-slipped using Vectashield mounting medium (H1000, Vector Laboratories, Peterborough, UK).

### 2.6. Quantification of Cells

Images of BrdU, Ki67, DCX-immunoreactive cells and double immunofluorescent positive cells were taken from Axio Scope 1 (Carl Zeiss, Germany) equipped with digital camera (Axiocam, Carl Zeiss) or fluorescent microscope (Olympus, BX-URA2). The cells in all groups were counted in 7 sections/each animal. For analysis, unbiased 2D stereology rules were applied, and cells were counted using an unbiased dissector (one per section), 500 µmx500 µm in size. To ensure unbiased sectioning, the first section of the hippocampus was taken at random, then every 5^th^ section afterwards [45]. The total cell count was obtained by averaging the counts from the sections taken from each animal.

### 2.7. Statistics

Student *t* test, One-way analysis of variance (ANOVA) and Two way repeated measure ANOVA followed by Bonferroni's post hoc test were used for statistical analysis using Prism (Graph-Pad Prism®, San Diego, CA). All values were expressed as mean ± SEM. A *p*-value of <0.05 was considered to be statistically significant.

## Results

### 3.1. Effect of Age on Cell Proliferation and Cell Survival in WT and APP/PS1 Animals

**Figure 1:**
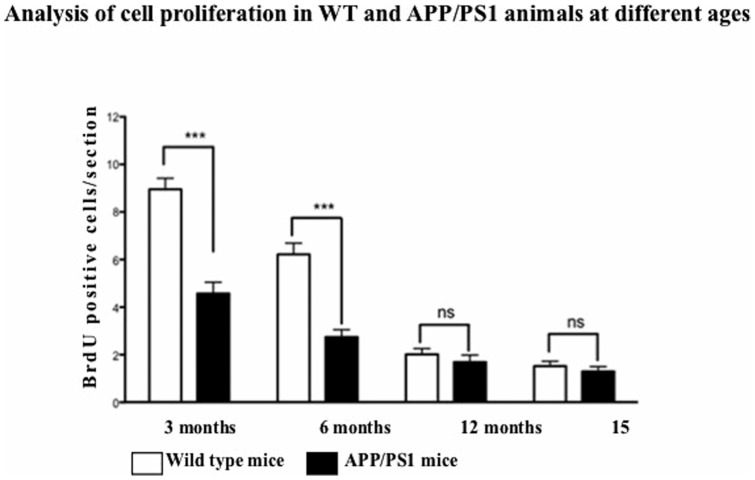
Quantification of BrdU positive cells in the SGZ of WT and APP/PS1 animals at different ages. Progenitor cell proliferation in SGZ decreases with age. Values are expressed as mean ± SEM (n = 6). ^***^p<0.001 compared with respective controls.

A significant decrease in number of BrdU positive cells was observed in both WT controls and APP/PS1 animals with progression of age (Figure 1). Significant effect of age (F = 97.13, df 3, p<0.001) and genotype (F = 69.14, df 1, p<0.001) was observed on cell proliferation, and the interaction between ageing and genotype was also significant (F = 17.89, df 3, p<0.001). Bonferroni post hoc tests revealed a difference in BrdU numbers in both WT and APP/PS1 mice from 3 to 6 months (t = 4.842, p<0.001 and t = 3.199, p<0.001, respectively), and 6 to 12 months (t = 6.486, p<0.001 and t = 2.354, p<0.001). No difference was seen between 12 to 15 months for both WT and APP/PS1 (t = 1.022, p>0.05 and t = 0.4442, p>0.05, respectively). Fewer BrdU positive cells were found in APP/PS1 mice compared to WT littermates at 3 and 6 months of age (t = 8.130, p<0.001 and t = 6.486, p<0.001). There was a non-significant trend for lower number of BrdU cells at 12 and 15 months in APP/PS1 mice compared to WT (t = 0.8885, p>0.05 and t = 0.3110, p>0.05). See Figure 1.

### 3.2 Acute Liraglutide Treatment (for 7 Days, Once Daily Ip.)

#### Cell Proliferation and Survival

Wild type mice treated with liraglutide did not show any significant increase in the number of newly generated cells in their dentate gyrus (BrdU stain), compared to animals treated with saline in 3, 6, 12 and 15 months of age (p>0.05). Treatment of APP*_swe_*/PS1_E9_ animals with liraglutide increases cell proliferation as compared to saline control. The 3 month old group showed a 33% increase. The 6 month group showed an increase of 41% (p<0.05), the 12 month group showed an increase of 69% (p<0.01) and the 15-month group showed a 65% increase in positive cells as compared to saline treated controls (p<0.05).

**Figure 2:**
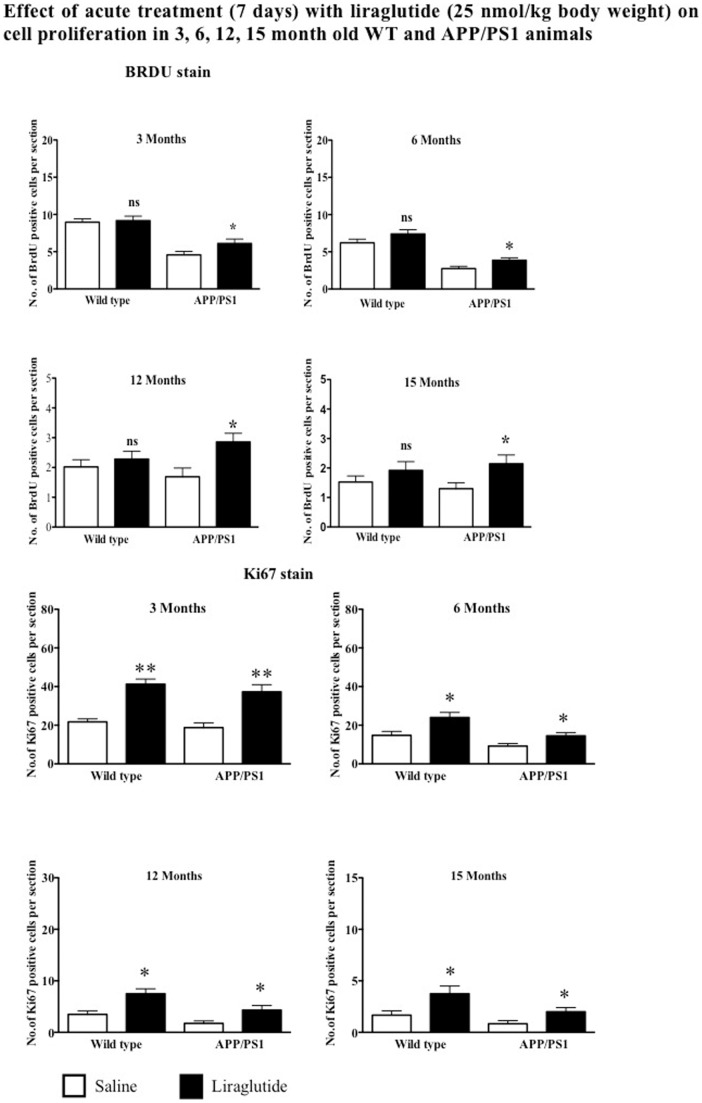
Quantification of BrdU positive cells in the SGZ. Acute treatment with liraglutide does not increase cell proliferation at any age in wild type animals whereas in APP/PS1 animals treatment with liraglutide increases cell proliferation regardless of age. Quantification of Ki67 positive cells in the SGZ. Acute treatment with liraglutide increases endogenous cell proliferation at all ages in wild type and APP/PS1 animals ^*^p<0.05, ^**^p<0.01 compared with saline control. Values are expressed as mean ± SEM (n = 6).

Increased cell proliferation as shown by Ki67 immunostaining was observed in WT animals after acute treatment with liraglutide. The 3 month old group showed 90% increase (p<0.01) in Ki67 cells in the DG compared to saline controls. The 6 months, 12 months and 15 months old WT animals showed 63%, 114% and 137% increase (p<0.05) in immunoreactive Ki67 cells respectively. Similarly, APP/PS1 animals of 3, 6, 12 and 15 months age showed 99%, 58%, 153% and 135% increase (p<0.01 and p<0.05) in Ki67 positive cells compared to saline treated groups respectively. See Figure 2.

#### Liraglutide Increases Neuroblast Differentiation in Both WT and APP/PS1 Animals

**Figure 3:**
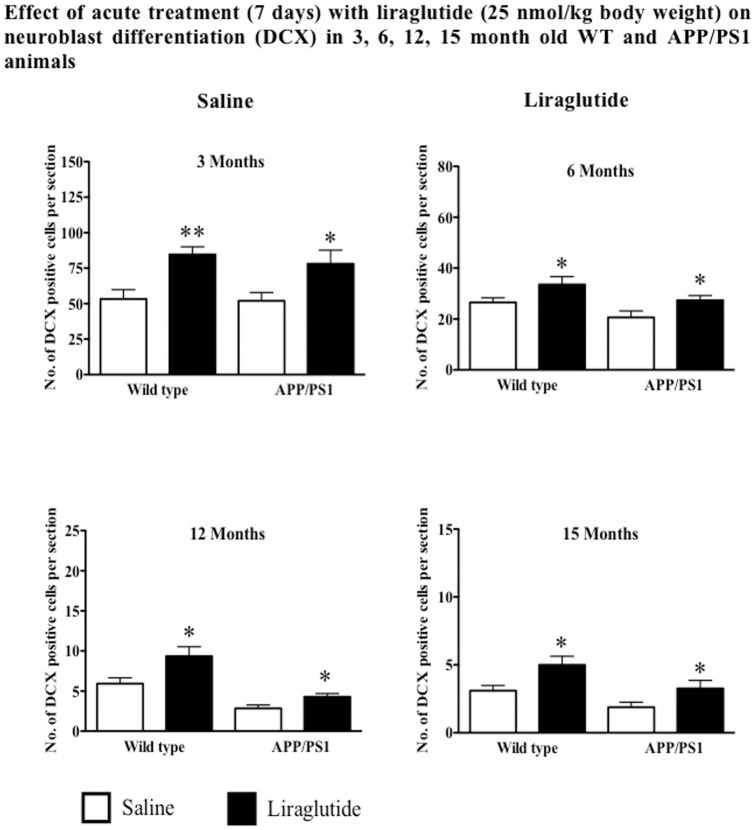
Acute treatment with liraglutide significantly increases DCX-immunoreactive cells in the DG of both wild type and APP/PS1 at all ages. ^*^p<0.05, ^**^p<0.01 compared with saline control. Values are expressed as mean ± SEM (n = 6).

Increased doublecortin positive cells were observed in 3, 6, 12 and 15 months old WT mice treated with liraglutide for 7 days by 59%, 26%, 57% and 61% respectively compared to saline treated groups (p<0.05 and p<0.01). Acute treatment of 3, 6, 12 and 15 months old APP/PS1 mice also showed increased DCX-immunoreactive cells by 50%, 33%, 53% and 72% respectively compared to control groups (p<0.05). See Figure 3.

#### Liraglutide Does Not Increase Neurogenesis in APP*_swe_*/PS1_E9_ and Wild Type Control

Double Immunostaining with BrdU/NeuN staining was utilised to evaluate the differentiation of progenitor cells to mature neuron. In acutely treated wild type animals, percentage difference of BrdU/NeuN co-labelled cells in drug group compared to saline control for 3-month was 8% (p>0.05), for 6-month was 21% (p>0.05), for 12-months was 20% (p>0.05) and for 15-month was 22% (p>0.05), no result was statistically significant. In AD mice, a non-significant trend was observed in where 3-month group showed 19% increase (p>0.05), for the 6 month group it was 18% (p>0.05), for the 12 month it was 21% (p>0.05) and for 15-month was 12% (p>0.05).

#### Gliogenesis in the Mouse Brain

Not all newly generated cells expressed the NeuN antigen, indicating they differentiated into other phenotypes like glia cells. Double immunostaining showed a very low percentage of newly expressed astrocytes and microglia in the dentate, and no difference was seen between groups. In Wild type animals that were acutely treated with liraglutide, the average percentage of newly produced GFAP and microglia cells were 5.68±3.15 and 6.3±2.65 respectively compared to 6.85±1.70 and 6.48±1.74 (p>0.05) in saline control animals. The percentages for GFAP and Iba1 was 7.3±3.3 and 7.55±1.47 respectively compared to 9.7±2.4 and 10.07±1.45 of those of saline controls which was not significant (p>0.05).

### 3.3. Chronically treated mice (37 days of injection once-daily ip.)

#### Liraglutide Increases Cell Proliferation and Survival in Wild Type Mice

**Figure 4:**
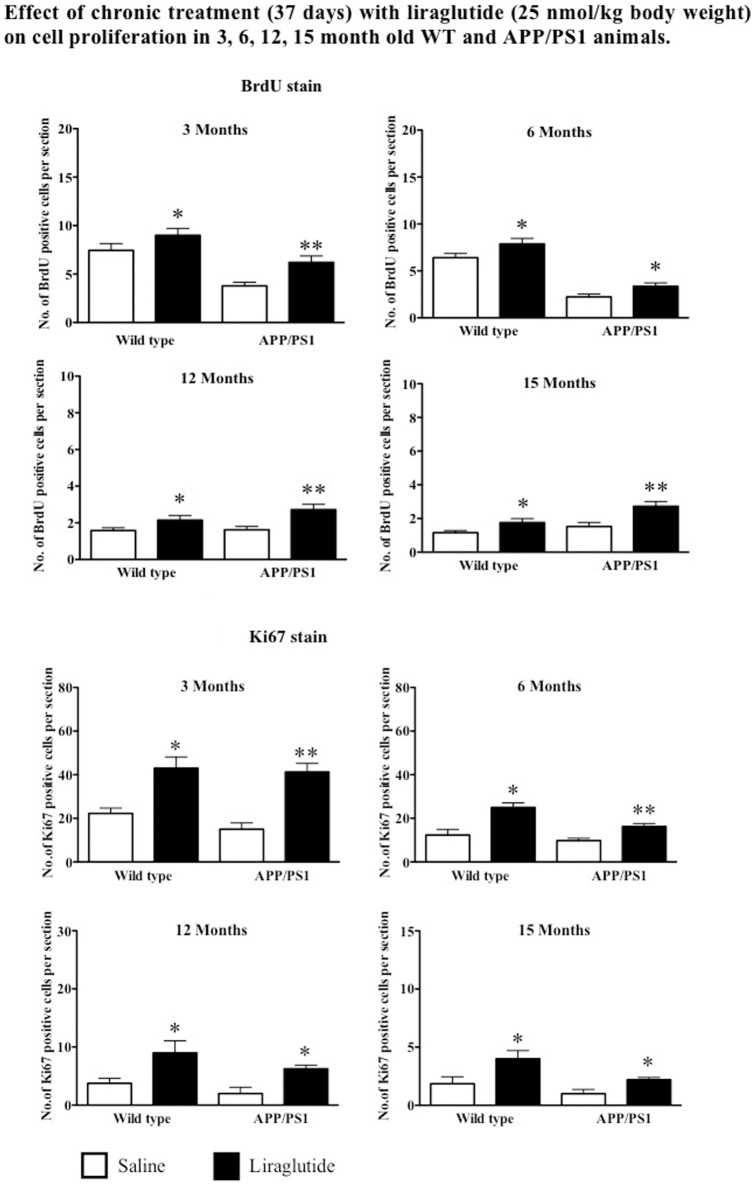
Quantification of BrdU positive cells in the SGZ. Chronic treatment with liraglutide increases cell proliferation in both wild type and APP/PS1 animals regardless of age. Quantification of Ki67 immunoreactive cells in the SGZ. Chronic treatment with liraglutide increases endogenous cell proliferation in both wild type and APP/PS1 animals regardless of age. ^*^p<0.05, ^**^p<0.01 compared with saline control. Values are expressed as mean ± SEM (n = 6).

All four age groups of wild type mice showed a significant increase in the number of BrdU positive cells in the DG as compare to saline controls. There was an increase of 20%, (p = 0.0457), 22%, (p = 0.0467), 36% (p = 0.0455) and 52% (p<0.05) of BrdU positive cell in mice group aged 3, 6, 12 and 15 months respectively**.** Drug treatment of APP*_swe_*/PS1_E9_ animals with liraglutide increases cell proliferation (BrdU stain) as compared to saline control. The 3 month old group showed a 55% increase (p<0.01). The 6 month group showed an increase of 50% (p<0.05), the 12 month group showed an increase of 68% (p<0.01) and the 15-month group showed a 79% increase in positive cells as compared to saline treated controls (p<0.01). See Figure 4.

When analysing Ki67 positive cells, liraglutide increased cell proliferation in 3, 6, 12 and 15 months old WT mice by 94%, 103%, 143% and 122% respectively when compared to saline treated group (p<0.05). Similarly, 3, 6, 12, and 15 months old APP/PS1 mice group also showed increased levels of Ki67 immunostaining by 175%, 70% 215% and 120% respectively compared to saline groups (p<0.01). See Figure 4.

#### Chronic Administration of Liraglutide Increases Neuroblast Differentiation in Both WT and APP/PS1 Animals

**Figure 5:**
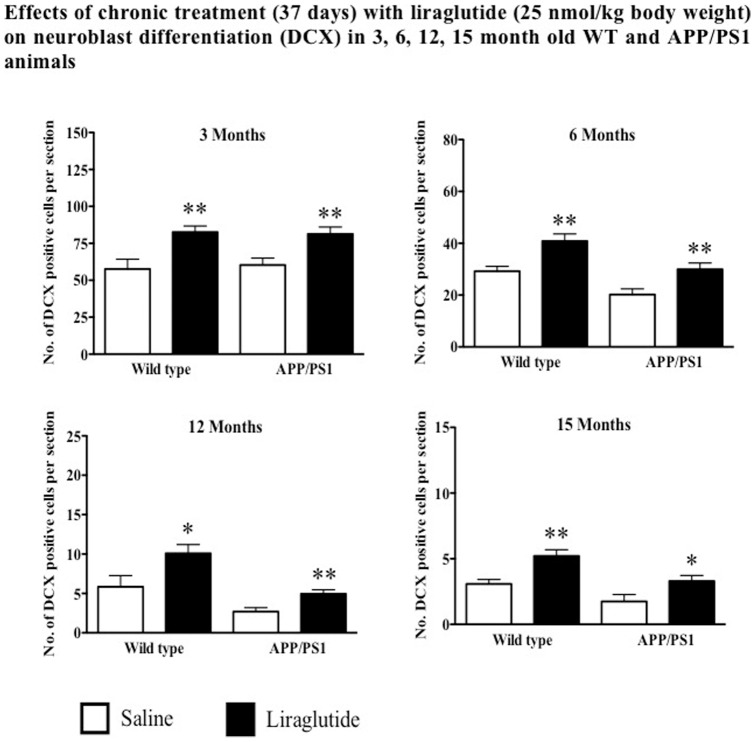
Chronic treatment with liraglutide increases neuroblast differentiation in both wild type and APP/PS1 animals regardless of age. ^*^p<0.05, ^**^p<0.01 compared with saline control. Values are expressed as mean ± SEM (n = 6).

Chronic treatment with liraglutide increased the numbers of immature neurons (DCX stain) in 3, 6, 12 and 15 months in WT mice by 43%, 40%, 74% and 68% respectively compared to control groups (p<0.05 and p<0.01). Similarly, increased levels of DCX positive cells in 3, 6, 12 and 15 months APP/PS1 animals by 35%, 48%, 88% and 94% respectively compared to saline groups (p<0.05 and p<0.01). See Figure 5.

#### Liraglutide Treatment Increases Neuronal Differentiation

**Figure 6:**
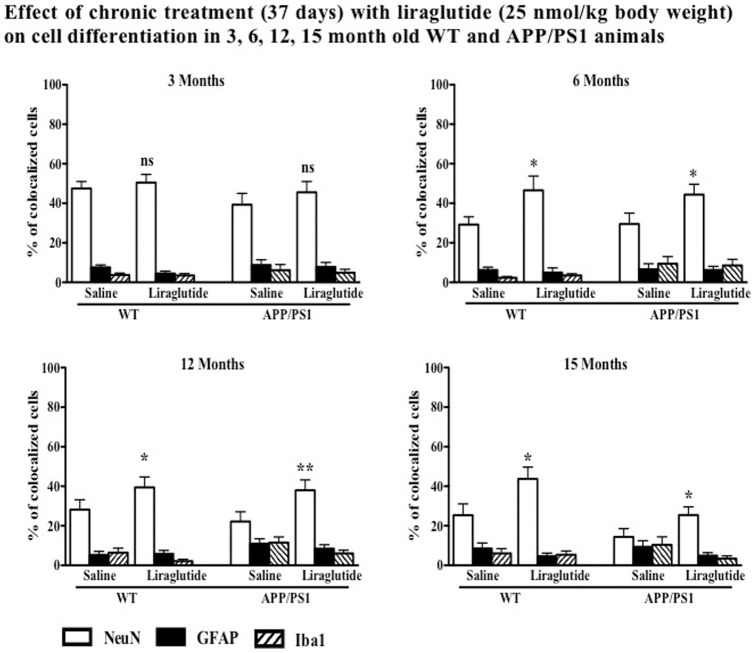
Chronic treatment with liraglutide significantly increases differentiation of newly produced cells to new neurons in wild type group at 6, 12 and 15 months wild type mice and APP/PS1 mice. No difference was seen at 3 months of age. No significant difference was seen in newly generated glia cell. ^*^p<0.05, ^**^p<0.01 compared with saline control. Values are expressed as mean ± SEM (n = 6).

The numbers of BrdU+NeuN (double staining) positive cells in WT and APP/PS1 animals treated with liraglutide compared to saline group are shown in Figure 6. In both wild type littermate and APP/PS1 mice, chronic treatment with liraglutide showed an increase in neuronal numbers compared to saline controls. The Wild type and APP/PS1 6-month group showed an increase of 57% (p<0.05) and 50% (p<0.05) of new neurons compared to saline control. For 12-months, the difference was 41% (p<0.05) and 70% (p<0.01), and for the 15-month group the difference was 72% (p<0.05) and 77% (p<0.05) respectively. No difference was seen in the 3-month old groups for both wild type (p>0.05) and APP/PS1 (p>0.05) animals (Figure 6).

#### Gliogenesis

Not all newly generated cells expressed the NeuN antigen, indicating they differentiated into other phenotypes like glia cells. Double immunostaining showed a very low percentage of newly expressed astrocytes and microglia in the dentate, and no difference was seen between groups. In chronically treated animals, the average percentage was 6.28±1.20 and 3.58±1.60 compared to 4.95±1.07 and 4.75±1.80 (p>0.05) respectively. In APP/PS1 animals the average percentage of GFAP and Iba1 was higher compared to wild type. The values for GFAP and Iba1 was 6.85±1.75 and 5.70±2.55 respectively compared to 8.85±2.0 and 9.38±2.65 of those of saline group, which was not significant (p>0.05). See Figure 6.

## Discussion

Generation of new progenitor cells and their differentiation into neurons in the dentate gyrus takes place throughout the life of rodents and humans [3], [11], [42]. The new neurons have shown to extend their axonal projections into the hippocampal CA3 area [46], indicating that they play a functional role in the neuronal network of the hippocampus.

There is an age-dependent decline in the number of immunoreactive BrdU cells, a marker of proliferating cells, in the SGZ of the dentate gyrus in both wild type and APP/PS1 animals. This decrease in neurogenesis with age has been reported previously on rodents and humans [10], [11], [42]. Significantly, APP/PS1 mice showed consistently lower numbers of newly generated cells in their DG compared to aged matched wild type littermates when compared at ages 3 months and 6 months, while at 12 and 15 months no difference was seen. This may be due to AD pathology impairing the proliferation of NSC at earlier stages of the life in the AD mouse model, which has been described previously in several studies with various mouse models of AD [19], [42], [47]. Also, as the numbers of new neurons were already very low in the older groups, a significant reduction in numbers would be hard to achieve.

The important roles of various factors such as exercise, enriched environment, trauma and growth factors on the regulation of neurogenesis have been studied in detail [1] [48], [49]. Growth factors such as epidermal growth factor (EGF), fibroblast growth factor-2 (FGF-2) and glia cell line-derived neurotrophic factor (GDNF) when administered intracerebroventricular (i.c.v.) in rats with ischemic brain injury, increased neurogenesis was observed, and the new neurons were shown to integrate functionally into the network [50], [51]. The roles of NGF and BDNF in neurogenesis also has been researched in some detail, and their potential use as a treatment for neurodegenerative disease has been investigated [17], [52]–[54]. Thus, compounds that can selectively promote proliferation of NSC and prompt their differentiation to neuronal phenotype may be of use as a treatment of neurodegenerative diseases such as AD [16].

We observed that a 1-week treatment of wild type animals with liraglutide had no effect on BrdU positive cell numbers, but showed increased endogenous cell proliferation as shown by Ki67 immunoreactive positive cells at 3, 6, 12 or 15 months of age. In contrast, APP/PS1 animals treated acutely for 7 days with liraglutide showed a significant increase in BrdU and Ki67 positive cells at ages 3, 6, 12 and 15 months. Amyloid deposition is related to decreased neurogenesis in AD mice. APP/PS1 double transgenic mice develop early-onset amyloidosis, as young as 3 months, with plaque deposits by 5–6 months, progressing with age up to 15 months [42], [55]. Beta-amyloid oligomers have been shown to interfere with insulin signaling [56], and since insulin is an important growth factor that also activates stem cells, this impairment in insulin signaling may be a reason for the impairment of stem cell proliferation found in the APP/PS1 mice [57], [58]. A second reason for the normalisation of neurogenesis by liraglutide may be the fact that the chronic inflammation response was reduced. Cytokines released during a chronic inflammation response reduced stem cell proliferation [21]. We have previously shown that liraglutide reduces the chronic inflammation response in this APP/PS1 mouse strain [35]. This would explain why there was little effect on neuronal progenitor proliferation in wild type mice as there were no plaques nor the associated chronic inflammation response. The effects of acute treatment by liraglutide on cell proliferation *in-vivo* in AD mice has not been studied previously.

The effects of long-term administration of liraglutide on progenitor cell proliferation in the SGZ of dentate gyrus was examined in APP/PS1 and WT littermates. The 37- day treatment with liraglutide significantly increased the number of immunopositive BrdU cells and also Ki67 positive cells in 3, 6, 12 and 15-months in both APP/PS1 and WT animals compared to saline controls. Previous studies showed that GLP-1 and analogues of GLP-1 induce cell proliferation *in vivo* and *in vitro* [9], [59]–[61]. GLP-1 receptors are expressed all throughout the brain especially on neurons in the hippocampus and cortex [33], [62]. Furthermore, GLP-1 also functions as a growth factor, has neuroprotective characteristics and enhances cell survival [22], [63]. GLP-1R mediated activation of mitogen activated protein kinase (MAPK) is crucial for hippocampal neurogenesis [38], which is associated with hippocampal-dependent learning and memory [64], [65].

Differentiation capacity of NSC on acute treatment with liraglutide showed no changes in both WT and APP/PS1 mice at any age. There was no difference in the percentage of newly generated cells expressing mature marker (NeuN) of neurons compared to control group. No difference in neuronal phenotype number was seen in 3-month old WT and APP/PS1 group treated chronically with liraglutide. Interestingly, in chronic treatment with liraglutide, both APP/PS1 and WT littermates showed significant increase in number of cells incorporating BrdU and mature neuronal marker NeuN at 6, 12 and 15 months, indicating increased neuroblast differentiation. Interestingly, both acute and chronic treatment increased neuroblast differentiation in WT and APP/PS1 animals. This suggest acute requirement of liraglutide is sufficient for its trophic properties in neuroblast differentiation but chronic administration is essential for full differentiation into mature neurons.

Not all newly generated cells differentiate into neuron, but some transform into glial phenotype. Liraglutide did not appear to affect the differentiation into glial. There was a trend towards a decrease the number of new cells transforming to glia cells, which was not significant and may be explained by an increase in cells differentiating into neurons. Previous studies have shown that liraglutide significantly decreases activated microglia and activated astrocytes in APP/PS1 mouse brains and WT mouse brains [35], but no research has been conducted previously on effects of incretins on differentiation of NSC into glial phenotypes. The results shown here suggest a chronic requirement of liraglutide to activate the differentiation process of NSC into neurons. However, our study does not rule out that an increased survival of NSC that may contribute to net neurogenic potential of liraglutide. Past studies have shown an increase in neuronal markers (MAP2, b-III-tubulin) in cell cultures and an increase in DCX positive cells in SVZ of rat brain after chronic exendin-4 treatment [9], [60]. Additionally, GLP-1 promotes beta cell proliferation [61] and differentiation from embryonic stem cells [66]. The mechanism of action of liraglutide in differentiation could be exerted via upregulation of Mash1 (involved in neuronal differentiation in hippocampus) [67].

This is the first study that investigated the proliferation and differentiation properties of GLP-1 analogue, liraglutide in wild type and APP/PS1 animals at different ages. We are also first to look at the effects of acute and chronic treatment with liraglutide on progenitor cell proliferation and differentiation at different ages of wild type and animals with AD pathology.

Based on findings of our study and previous work that showed liraglutide improves cell proliferation in SGZ, increases differentiation of progenitor cells to neurons, this drug and other GLP-1 analogues have therapeutic potential to treat neurodegenerative disorders such as Alzheimer's and Parkinson's disease.
